# HPV-Negative Oral Squamous Cell Carcinoma Arising from Oral Submucous Fibrosis with p16INK4A Positivity and Cellular Senescence: A Case Report

**DOI:** 10.4317/jced.62698

**Published:** 2025-04-01

**Authors:** Akhilanand Chaurasia, Pavlos Pantelis, Giorgos Theocharous, Dimitris Veroutis, Athanassios Kotsinas, Eva V. Gorgouli, Eleni Georgakopoulou

**Affiliations:** 1 Department of Oral Medicine and Radiology, King George Medical University, India; 2 Molecular Carcinogenesis Group, Department of Histology and Embryology, School of Medicine, National and Kapodistrian University of Athens, Athens, Greece; 3 Oral medicine private Practice, 4 Fokaias str 14232, Athens, Greece

## Abstract

**Background:**

Oral Submucous Fibrosis (OSF) is a chronic, progressive, and potentially malignant disorder primarily associated with areca nut chewing. While Oral Squamous Cell Carcinoma (OSCC) typically develops in the tongue and floor of the mouth, its occurrence in the buccal mucosa in the context of OSF is less common. The molecular mechanisms underlying OSF-associated OSCC remain unclear. p16INK4A is widely recognized as a surrogate marker for HPV-driven carcinogenesis; however, its role as an indicator of cellular senescence is increasingly acknowledged. Given that senescence contributes to various pathologies, including cancer, this case report explores its potential role in the pathogenesis of OSF-associated OSCC.

**Case Presentation:**

We present the case of a 39-year-old male with a 10-year history of gutkha chewing, who developed clinically advanced OSF and a well-differentiated OSCC of the buccal mucosa. Immunohistochemical (IHC) analysis revealed strong p16INK4A expression, increased p21WAF1/cip1 levels, and low Ki67 proliferative activity. Notably, Polymerase Chain Reaction (PCR) testing for HPV was negative. Further staining with SenTraGor (GL13) confirmed the presence of senescent cells, suggesting that p16INK4A overexpression in this case reflects cellular senescence rather than an HPV-driven oncogenic process.

**Conclusions:**

This case highlights the necessity of thorough molecular assessment when interpreting p16INK4A positivity in OSF-associated OSCC. The HPV-negative status, coupled with senescence markers, suggests that p16INK4A expression in such cases may show a senescence-associated tumorigenic pathway rather than HPV-mediated carcinogenesis. These findings support the inclusion of cellular senescence markers like SenTraGor in the diagnostic evaluation of OSCC arising from OSF.

** Key words:**Oral Submucous Fibrosis, OSCC, p16INK4A, Cellular Senescence, HPV-Negative, Buccal Mucosa, SenTraGor.

## Introduction

Oral Submucous Fibrosis (OSF) is a chronic, progressive, and debilitating fibrotic disorder that significantly increases the risk of malignant transformation into Oral Squamous Cell Carcinoma (OSCC) (1). While OSCC commonly arises in the tongue and floor of the mouth, the buccal mucosa is also a frequently affected site, particularly in patients with OSF, where carcinogenesis is strongly linked to areca nut and its derivatives (gutkha, pan masala) (2). This etiological association is particularly prevalent in Southeast Asia, where habitual areca nut chewing is widespread.

In HPV-positive head and neck cancers, p16INK4A overexpression is a well-established surrogate marker of viral oncogenesis, reflecting the disruption of the retinoblastoma (Rb) pathway by HPV oncoproteins (3). However, p16INK4A is also a recognized biomarker of cellular senescence—a state of irreversible cell cycle arrest induced by persistent DNA damage, oxidative stress, and chronic inflammation (4,5). While cellular senescence initially serves as a protective mechanism by preventing uncontrolled cell proliferation, its prolonged presence can contribute to tumor progression and recurrence in malignancies (6).

This report presents a case of OSCC arising in the buccal mucosa of a young patient with clinically advanced OSF. Despite strong p16INK4A expression, HPV testing was negative, suggesting that p16INK4A upregulation in this case reflects a senescence-driven rather than a virus-mediated oncogenic process. The findings emphasize the importance of integrating senescence markers into the molecular assessment of OSCC, particularly in OSF-associated malignancies.

## Case Report

Clinical History and Examination

A 39-year-old male presented with progressive trismus, persistent oral burning sensation, and a non-healing ulcer on the left buccal mucosa. The patient reported a 10-year history of gutkha chewing but denied smoking or alcohol use.

On clinical examination, the patient showed severe trismus, with a restricted mouth opening of approximately 20 mm inter-incisally. The left buccal mucosa showed an ulcerative lesion measuring approximately 1.5 cm, with indurated margins. The surrounding mucosa was firm, blanched, and exhibited palpable fibrotic bands, consistent with OSF. A biopsy was performed, revealing dense submucosal collagen deposition and fibrosis characteristic of OSF, along with invasive nests of keratinizing squamous epithelial cells infiltrating the submucosa, confirming the diagnosis of OSCC.

**Figure 1:**
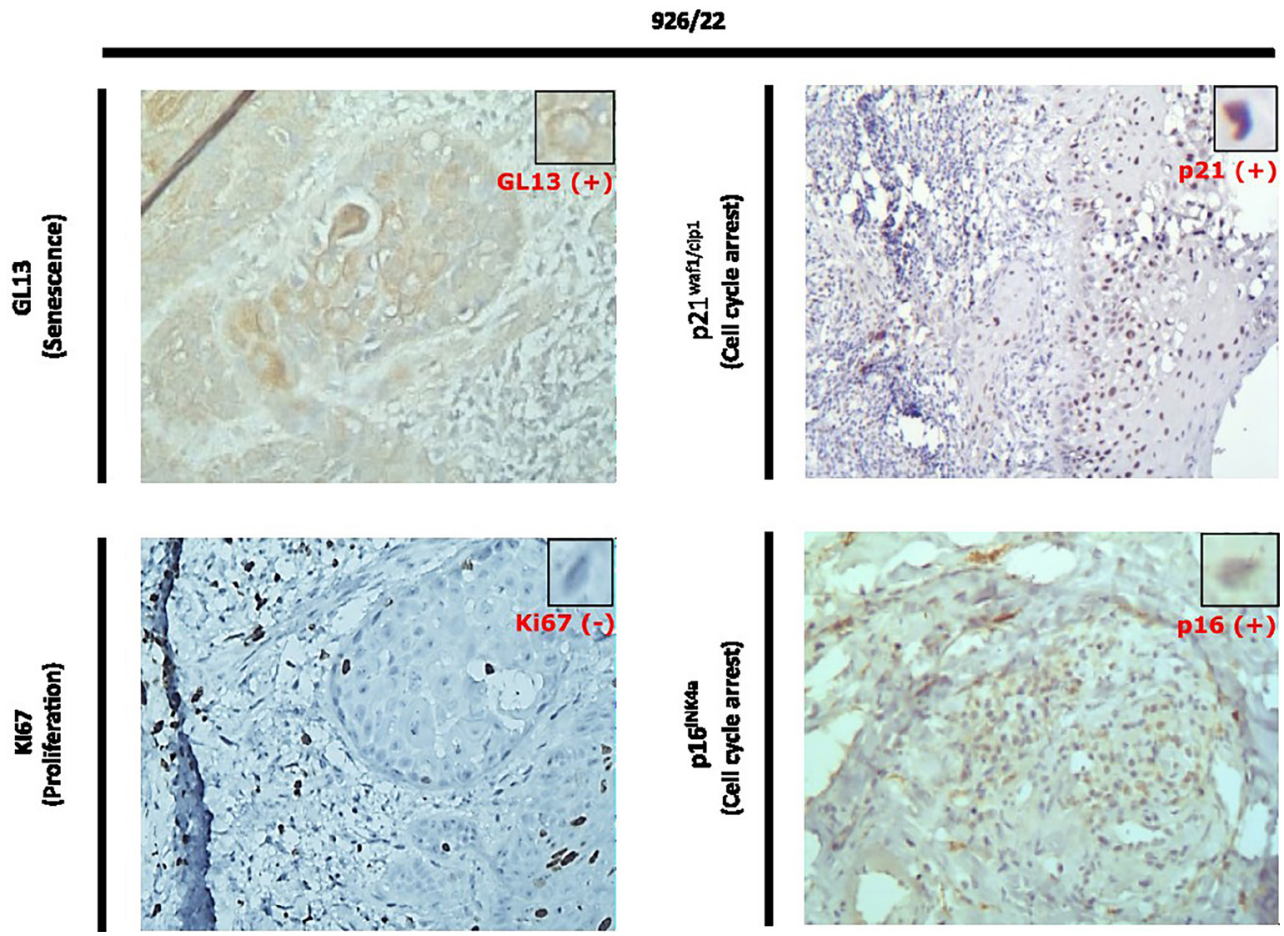
IHC and SenTraGor (GL13) stains proving relationship between p16 positivity and cellular senescence (colocalization of markers).

Immunohistochemical (IHC) analysis demonstrated strong nuclear and cytoplasmic positivity for p16INK4A in the tumor cells, along with upregulation of p21WAF1/Cip1 and minimal Ki67 activity, indicative of reduced proliferative capacity. Polymerase Chain Reaction (PCR) testing for high-risk HPV types 16 and 18 was negative, ruling out a viral etiology for the observed p16INK4A overexpression (Fig. 1).

To assess whether cellular senescence contributed to the disease pathogenesis, tissue sections were stained with SenTraGor (GL13), a senescence-specific biomarker that detects lipofuscin accumulation in accordance with established guidelines for senescence detection (7,8). The staining revealed positivity in dysplastic epithelial regions, confirming the presence of senescent cells. These regions exhibited negligible Ki67 co-expression, further supporting a state of irreversible cellular growth arrest, consistent with senescence-driven rather than HPV-mediated oncogenesis (Fig. 1) (9).

The patient underwent surgical resection followed by adjuvant radiotherapy. He remained disease-free for two years but later succumbed to an unrelated cause.

## Discussion

p16INK4A Positivity in a Young Male with HPV-Negative OSCC: A Closer Look at Cellular Senescence

OSCC in young male patients, is often linked to high-risk HPV infection (3). Given the established association between p16INK4A overexpression and HPV-driven carcinogenesis, a first suspicion of HPV-related malignancy was warranted in this case. However, PCR testing for high-risk HPV types 16 and 18 was negative, prompting further investigation into alternative mechanisms underlying p16INK4A upregulation.

**Figure 2:**
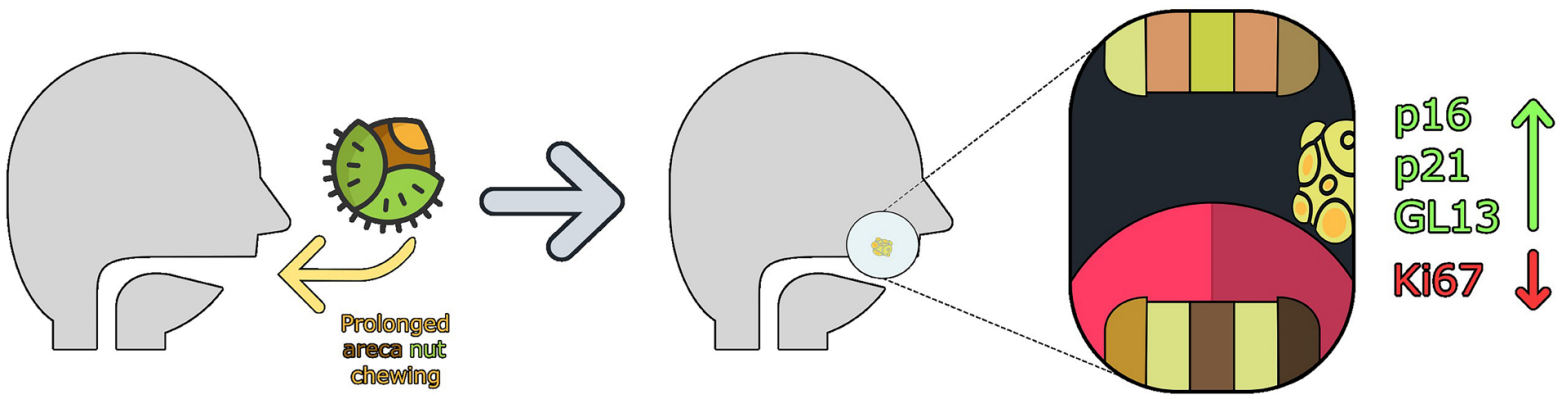
Our Hypothesis of how this case supports the effect of cellular senescence in OSF progression to OSCC.

Recognizing the patient’s history of prolonged areca nut exposure and the presence of extensive fibrosis, we explored the role of cellular senescence as a contributing factor. Senescent cells, despite being growth-arrested, exert profound effects on the tumor microenvironment through the Senescence-Associated Secretory Phenotype (SASP), which promotes chronic inflammation and can facilitate tumor progression (10). In this case, the positivity for p16INK4A was accompanied by increased p21WAF1/Cip1 expression, minimal Ki67 activity, and SenTraGor staining positivity, collectively supporting a senescence-driven rather than an HPV-mediated oncogenic pathway (this hypothesis is summarized in Fig. 2).

Clinical and Molecular Considerations in OSF-Related OSCC

This case underscores the importance of considering cellular senescence in OSCCs arising in OSF, particularly in young patients where an HPV-positive etiology is frequently assumed. The presence of a dense fibrotic microenvironment in OSF leads to sustained oxidative stress and chronic inflammation, key drivers of senescence induction (6). The findings of this case suggest that p16INK4A expression in OSCC is not always synonymous with HPV infection and highlight the necessity of complementary molecular assessments, such as senescence-specific markers, to accurately interpret its clinical significance.

Therapeutic Implications and Future Directions

Targeting senescent cells, a concept known as senolysis, represents an emerging approach in oncology. While several senolytic agents have been identified, many are limited by off-target effects and toxicity. Recent advancements in senolytic platforms, such as mGL392, which utilizes a lipofuscin-binding domain conjugated with dasatinib, have shown promise in selectively eliminating senescent cells (11). Future research is needed to evaluate the potential therapeutic benefits of senolysis in OSF-associated malignancies, particularly in preventing malignant transformation and recurrence.

## Conclusions

This case highlights the need for a nuanced interpretation of p16INK4A positivity in OSF-associated OSCC. The negative HPV status, coupled with senescence-specific markers, suggests that p16INK4A overexpression in this setting reflects a senescence-driven rather than a virus-mediated oncogenic process. Further research into the interplay between fibrosis, inflammation, and senescence in OSCC may provide novel insights into targeted interventions for OSF-related malignancies.

## Data Availability

The datasets used and/or analyzed during the current study are available from the corresponding author.
